# Treatment Options for Metaplastic Breast Cancer

**DOI:** 10.5402/2012/706162

**Published:** 2012-06-21

**Authors:** Dhruvil R. Shah, Warren H. Tseng, Steve R. Martinez

**Affiliations:** Division of Surgical Oncology, UC Davis Comprehensive Cancer, Sacramento, CA 95817, USA

## Abstract

Metaplastic breast cancer (MBC) is a malignancy characterized by the histologic presence of two or more cellular types, commonly a mixture of epithelial and mesenchymal components. MBC is rare relative to invasive ductal carcinoma (IDC), representing less than 1% of all breast cancers. Other than a lower rate of lymph node metastases, MBC tumors display poorer prognostic features relative to IDC. Due to its low incidence and pathological variability, the ideal treatment paradigm for MBC is unknown. Because of its rarity, MBC has been treated as a variant of IDC. Despite similar treatment regimens, however, patients with MBC have worse outcomes. Recent research is focused on biological differences between MBC and IDC and potential novel targets for chemotherapeutic agents. This paper serves as a summation of current literature on approaches to the multidisciplinary treatment of patients with MBC.

## 1. Introduction

Metaplastic breast cancer (MBC) is a rare malignancy characterized by the histologic presence of two or more cellular types, commonly a mixture of epithelial and mesenchymal components [1–6]. MBC represents 0.25–1% of breast cancers diagnosed annually [1, 7, 8]. Due to its relative rarity and heterogeneous histologic presentation, the pathologic diagnosis of MBC is difficult. The World Health Organization (WHO) recognized MBC as a unique pathologic entity in 2000. Since then, the incidence of MBC has risen, likely representing an increased recognition by pathologists [8, 9]. Overall, greater than 70% of patients with MBC present with American Joint Committee on Cancer (AJCC) stage II or greater disease as compared to approximately 50% of patients with IDC [8]. Compared to patients with IDC, those with MBC have worse outcomes with 5-year survival rates ranging from 49% to 68% [1, 10, 11].

The optimal treatment strategies for MBC are unknown. Management of MBC has largely paralleled that of IDC, despite growing evidence that MBC is a distinct entity that lies along the spectrum of basal-like breast cancers. This paper serves as a summation of current literature and approaches to the multimodality treatment of patients with MBC.

## 2. Surgical Therapy

The surgical treatment of MBC has largely paralleled that of IDC. With the publication of NSABP B-06 trial results, the surgical approach to IDC shifted from mastectomy to breast conservation therapy for appropriate patients. Large tumors (≥5 cm) are a relative contraindication to breast conservation therapy [12] and even less extensive tumors may preclude breast conservation in smaller-breasted patients. These guidelines are particularly important for MBC patients, as they typically present with larger tumors compared to their IDC counterparts [8, 13]. As one might expect, relative to those with IDC, a higher percentage of patients with MBC receive mastectomy rather than lumpectomy [8, 14].

Despite the larger tumor size at presentation, MBC histology should not preclude breast conservation therapy in appropriate patients. Tseng and Martinez found no difference in overall or disease-specific survival whether MBC patients were treated with mastectomy or lumpectomy, even after controlling for known prognostic factors [13]. Similarly, Dave et al. found no difference in overall or disease-free survival between patients with MBC undergoing either modified radical mastectomy or breast conservation therapy [15].

Lymph node staging of patients with MBC is evolving, reflective of the changes in approach to axillary staging of patients with IDC. Axillary staging had traditionally been performed with axillary lymph node dissection (ALND). However, sentinel lymph node biopsy (SLNB) has largely replaced ALND due to lower associated morbidity with similar accuracy in the detection of regional metastasis [16]. Prior to the American College of Surgeons Oncology Group (ACOSOG) Z0011 trial, all patients with a positive SLNB subsequently went on to receive a completion ALND. However, based on the results of this trial, completion ALND may not be indicated in a select group of women with early stage breast cancers undergoing lumpectomy and subsequent whole breast radiation [17]. The Z0011 trial did not address MBC patients as a particular subgroup, however. Therefore, data regarding the sensitivity and specificity of sentinel lymph node biopsy among MBC patients are unknown, as are data regarding the need for completion ALND for patients with sentinel lymph node metastasis. Several studies have demonstrated lower rates of axillary lymph node involvement among patients with MBC relative to IDC [8, 13]. Tseng and Martinez reported axillary lymph node involvement in 22% of MBC patients [13]. Pezzi et al. also demonstrated axillary lymph node metastases in 22% of those with MBC versus 34% of those with IDC and further noted that nodal positivity was more common among the carcinosarcoma variant [8]. Despite this, Beatty et al. demonstrated that SLNB and ALND are used equally in patients with MBC relative to women with IDC [18]. Tseng et al. showed that women with locally advanced MBC (i.e., primary tumors >5 cm and/or four or more metastatic axillary nodes) who underwent postmastectomy radiation therapy had improved survival relative to those not receiving such radiation [13]. Because completion ALND is often the only way to document ≥4 lymph node metastases and because patients undergoing mastectomy were not included in the ACOSOG Z0011 trial, it would be prudent to recommend completion ALND for MBC patients undergoing mastectomy if they have documented sentinel lymph node metastasis.

## 3. Chemotherapeutic and Hormone Therapy Approaches

There are little data that standard breast cancer chemotherapy regimens utilized for IDC are effective for women with MBC. Compared to stage-matched women with IDC, those with MBC receiving adjuvant chemotherapy have poor survival. Nevertheless, 33%–86% of MBC patients receive chemotherapy and are, in fact, twice as likely to receive chemotherapy as similarly matched patients with IDC [8, 11, 19]. Single institution retrospective studies and genomic profiling suggest that these tumors are largely chemoresistant [19]. A report from the Mayo clinic detailed 27 patients treated at their center over 20 years in which 33% received chemotherapy. Ten different regimens were used, resulting in one partial response [11]. This resistance to chemotherapies is likely a product of the complex genetic and nongenetic mechanisms within MBC that result in phenotypically diverse subclones and intratumoral heterogeneity. 

Hormonal therapy is just as ineffective as chemotherapy, and generally has no role in the management of patients with MBC. There is a high incidence of hormone receptor negativity as well as lower Her-2/neu overexpression in MBC [20]. MBCs are often classified along the spectrum of basal-type breast cancers of which 75–85% are triple negative (estrogen receptor, progesterone receptor, and Her-2/neu overexpression negative) [21, 22]. Patients with triple-negative MBC have poor 3-year disease-free survival compared to a similar group of triple-negative IDC patients receiving identical chemotherapy regimens [20]. 

Variations in histologic types may partially account for the observed resistance to standard IDC chemotherapeutic regimens as well as poorer survival. A study by Tseng et al. included tumors with heterogeneous histologic diagnoses representing histologic subtypes including metaplastic carcinoma not otherwise specified, carcinosarcoma, malignant myoepithelioma, adenosquamous carcinoma, epithelial-myoepithelial, and adenocarcinoma with squamous/cartilaginous/and spindle cell metaplasia [13]. These histologic subtypes were consistent with those described by Wargotz et al. [2–6], who subcategorized MBC into five types: matrix producing, spindle like, squamous with ductal origin, metaplastic with osteoclastic giant cells, and carcinosarcoma. In the analysis by Tseng et al., carcinosarcoma was associated with both poorer overall survival (HR 1.52, CI 1.13–2.04, *P* = 0.005) and disease-specific survival (HR 1.63, CI 1.16–2.31, *P* = 0.005) in multivariate analysis. These findings likely reflect biological differences between the nonepithelial sarcomatous elements found in carcinosarcoma as compared to the nonepithelial components of the other histological variants. 

Recent studies have investigated receptors that may potentially serve as novel targets for chemotherapy regimens. One such target is EGFR (Her-1). Leibl and Moinfar [23] examined 20 different MBC samples (8 heterogeneous elements, 7 spindle cell, 4 carcinosarcoma, and 1 matrix-producing) with immunohistochemical staining for the four members of the EGFR/Her family. They found that 14 of the 20 samples were positive for Her-1 whereas only 1 sample was positive for Her-2, an inverse finding to that of IDC. This would suggest that targeted protein kinase inhibitors such as gefitinib might be effective for some patients with MBC [23]. Clinical trials are needed to investigate these potential new therapies.

Likewise, other novel strategies have emerged to target the nonepithelial component of MBC tumors. One such strategy is the use of ifosfamide and etoposide for carcinosarcoma variants of MBC [24]. Hennessy et al., in an evaluation of sarcomatoid MBC patients treated at a single institution, reported no recurrence in the 3 patients that received doxorubicin and ifosfamide treatment [19]. 

## 4. Radiation Therapy

Information regarding the role of adjuvant radiation therapy (RT) for the treatment of MBC is sparse [15, 25]. In a series of 43 patients with MBC, Dave et al. [15] reported a 10.5% rate of local recurrence for patients receiving lumpectomy and adjuvant radiation. Total radiation consisted of 50–66 Gy with use of tangential fields and additional anteroposterior supraclavicular fields when regional nodes were treated. 

Results of the Tseng et al. analysis utilizing the SEER database suggested that adjuvant radiation improved both overall and disease-specific survival for all patients undergoing treatment for MBC, regardless of the type of operation performed (lumpectomy versus mastectomy). Patients receiving RT demonstrated 36% and 26% decreases in death from any cause and breast-related mortality, respectively. Results of multivariate analyses excluding patients with metastatic disease paralleled these findings, demonstrating 38% and 34% decreases in death from any cause and breast-related mortality, respectively [13]. This is in accordance with a meta-analysis demonstrating that for prevention of local recurrences will improve overall and disease-specific survival [26]. 

 Postmastectomy RT has a more limited role. In this setting, RT is recommended to patients with four or more metastatic axillary nodes, large (≥5 cm) primary tumors and chest wall invasion [27–29]. Tseng et al. described mastectomy patients who had received RT demonstrating a 33% decreased risk of death from any cause. Subgroup analysis of “high” and “normal” risk patients undergoing mastectomy demonstrated that specifically, patients undergoing mastectomy with tumors ≥5 cm or four or more metastatic axillary lymph nodes derived a 47% and 42% decreased risk of death from all-cause and breast-related mortality, respectively. Patients undergoing mastectomy with tumors ≤5 cm and less than 4 metastatic axillary lymph nodes, however, derived no benefit from RT [13]. This data would suggest that RT should be considered as a component of multimodality therapy for MBC patients undergoing mastectomy with these advanced features. 

## 5. Conclusion

Due to its rarity and heterogeneity, there is no “standard” therapy for all patients with MBC. Surgical treatment and axillary staging parallel that of IDC with breast conservation therapy being appropriate for a select group of patients. Traditional chemo- and hormonal therapies for IDC are ineffective against MBC and often associated with poorer survival, while histology specific novel chemotherapeutic strategies may offer a survival advantage. Targeted therapies based on individualized gene profiling, while promising for the future, are not commonly utilized. Finally, adjuvant radiation, regardless of the type of surgery, should be considered part of the multimodality therapy for patients with MBC. Clinical trials comparing standard therapies for IDC in patients with MBC are needed, but are unlikely to be accomplished due to the rarity of the disease.
